# Exploring the Relationships between Resilience and Turnover Intention in Chinese High School Teachers: Considering the Moderating Role of Job Burnout

**DOI:** 10.3390/ijerph18126418

**Published:** 2021-06-13

**Authors:** Fei Liu, Huaruo Chen, Jie Xu, Ya Wen, Tingting Fang

**Affiliations:** 1 School of Education Science, Nanjing Normal University, Nanjing 210046, China; 190601009@njnu.edu.cn (F.L.); 190601022@njnu.edu.cn (J.X.); 2 School of Teacher Education, Huaiyin Normal University, Nanjing 210046, China; 3 Center for Research and Reform in Education, Johns Hopkins University, Baltimore, MD 21286, USA; 4 School of Teacher Education, NanJing XiaoZhuang University, Nanjing 210046, China; ywen1133@126.com; 5 School of Psychology, Nanjing Normal University, Nanjing 210046, China; 192302037@njnu.edu.cn

**Keywords:** Chinese high school teachers, resilience, job burnout, turnover intention, COVID-19

## Abstract

Background: With the outbreak and spread of the COVID-19 epidemic, online teaching time has been extended continuously. The changes in teaching methods, teaching conditions, and teaching environment have brought great pressure and difficulties in adjustment to teachers, which have led to a series of physical and mental problems such as negativity, lack of confidence, and depression. The long-term accumulation of these problems makes teachers’ turnover intention increasingly serious. Methods: Based on these premises, this study took 449 high school teachers in China as research objects and investigated the relationship between high school teachers’ resilience, job burnout, and turnover intention in the context of the COVID-19 epidemic. Results: The resilience of high school teachers had a significant negative predictive effect on job burnout and turnover intention (r = −0.473, *p* < 0.05; r = −0.283, *p* < 0.05), while job burnout had a significant positive predictive effect on turnover intention (r = 0.485, *p* < 0.05). At the same time, job burnout played a moderating role between resilience and turnover intention (λ = −0.019, *p* < 0.001). Discussion: This study suggests that society, schools, families, and individuals should adopt various strategies to improve teachers’ adaptability and relieve teachers’ job burnout, so as to solve the practical problem of teachers’ high turnover intention and ensure continuous improvement and healthy development of online teaching.

## 1. Introduction

With the outbreak of COVID-19 in 2020, many changes have taken place in the field of school education in China and even around the whole world, such as the shift from traditional offline learning to online learning [1]. In the face of the impact of online teaching, students’ interest in learning has been reduced due to the lack of scene sense, which requires teachers to mobilize more resources to stimulate students’ interest in learning and ensure the teaching effect [2,3]. Furthermore, it is necessary for teachers to consider the normal operation of online equipment to ensure normal communication between teachers and students in teaching [4,5,6]. Homework correction and feedback also need to be carried out online, meaning that teachers need to master online teaching technology quickly and comprehensively [7,8]. More importantly, teachers must not only be responsible for the education of students but also deal with the problems in students’ daily lives, which means that they have no time to take care of personal problems, such as ill parents. This situation undoubtedly increases teachers’ physical and mental pressure [9]. In brief, with the continuous outbreak of COVID-19 and the lengthening of online teaching time, many problems in education have become more apparent: teachers’ psychological bearing capacity has been affected, job burnout has gradually increased, and some teachers’ turnover intentions may have been further amplified [10,11,12]. In order to better cope with this situation, teachers need to strengthen their ability to adapt to changes [13]. Therefore, it is of great significance to explore the influence of teachers’ resilience and job burnout on turnover intention in the current situation in which the global coronavirus epidemic is spreading and intensifying.

Resilience refers to the phenomenon in which individuals show positive psychological development after maternal love deprivation. However, due to the different research fields and objects involved, the definition of resilience has not achieved uniformity up to now [14]. Generally speaking, resilience refers to the good adaptation of individuals in the face of setbacks, difficulties, or other major life pressures and is not only a dynamic process but also a positive psychological quality [15]. Oswald (2003) introduced resilience into the teacher evaluation project and put forward the concept of teacher resilience for the first time [16]. Teachers in the field of education should not only face the challenges and pressures brought by competition in the workplace [17] but also deal with the setbacks and troubles in the growth of teenagers [18]. Especially in the last ten years, teachers have been faced with various pressures from schools and families, resulting in varying degrees of job burnout and even resignation [19,20]. This requires teachers to have better adaptability [21]. Job burnout, defined as emotional exhaustion, personality disintegration, and personal achievement decline syndrome, is regarded by the World Health Organization as a medical disease, a career-related problem [22], and a tense interpersonal reaction in the workplace [23]. Previous research has found that professionals who provide social and humanistic services (including teachers from different teaching fields) often experience job burnout [24,25,26]. In the educational context, many factors can induce job burnout syndrome, mainly individual physiological and psychological factors, communicative relations, and environmental factors [27]. Relevant research has shown that teachers’ job burnout is caused by their long-term and extensive job stress, and its main symptoms are tension and fatigue in job perception, excessive obedience in the face of school and family, and low personal accomplishment [28]. As an important group providing K-12 education, there is plenty of empirical evidence that job burnout is a common problem among high school teachers [29,30]. Turnover intention has been put forward against the phenomenon of voluntary turnover and refers to the intentions of workers to leave a specific organization after working for a period of time and after some consideration [31]. Turnover intention is not equal to turnover behavior [32]. Turnover intention represents a certain possibility, while turnover behavior is a fact that does happen [33]. In view of the fact that it is difficult to measure turnover behavior, it is more meaningful to study turnover intention than other variables in the study of antecedent variables of turnover behavior, and turnover intention is usually taken as a substitute variable of turnover behavior [34]. Previous research has proved that the strongest and most direct variable to predict employee turnover is turnover intention, this being the most effective indicator for the prediction of actual turnover behavior [35]. As previous research has proved that the departure of teachers is a great loss to schools [36,37], studying teachers’ turnover intention is of great significance for schools to retain talents who can provide high-quality basic education.

With the deepening of the research on teachers’ professional development, it is hardly surprising that the relationship between resilience, job burnout, and turnover intention has attracted considerable attention in recent years. Much of the earlier work emphasized the relationship between two of the variables but rarely emphasized all three variables [38]. Therefore, in this study we classified and described the previous studies as follows. Firstly, previous research on turnover intention was found to focus on lacking self-ability, whereas researchers have increasingly shifted attention to self-adaptation and resilience after in-depth research. For example, Bowles and Arnup (2016) pointed out that teachers’ attrition is widely considered to have a negative impact on students’ achievements and schools’ development and that resilience is an important factor to effectively predict turnover intention [39]. Smith et al. (2020) suggested that resilience has a significant indirect negative association with turnover intention [40]. Secondly, job burnout, as a state produced by individuals at work, is considered an important factor in the study of turnover intention [41]. Schaack and Stedron (2020) used a two-level mediation model to explore the relationship between job burnout and turnover intention that showed that teachers’ lower wages lead to more emotional exhaustion and that those who lack a common vision with their organizations are likely to express their intention to leave [42]. Finally, some previous research has found that resilience plays a partial mediating role between job burnout and turnover intention in bank employees [43,44,45]. Tong and Wang (2015) showed that job burnout plays a partial mediating role between turnover intention and resilience [46]. Although most research up to now has involved the interactions between resilience, job burnout and turnover intention, the mechanisms of action at play are different and there is no unified statement to describe them. Particularly, there has been little research on the relationship between resilience, job burnout, and turnover intention in the study of teachers’ professional problems generally, let alone in the current epidemic situation [47]. Therefore, the main purpose of this study was to measure the influences of resilience and job burnout on turnover intention and explore the mechanisms at play with regard to high school teachers with the heaviest teaching tasks during the height of the COVID-19 epidemic. This study raises the following questions:

- Question 1. Does resilience significantly negatively predict turnover intention?
- Question 2. Does job burnout significantly positively predict turnover intention?
- Question 3. Does job burnout play an intermediary role between resilience and turnover intention?

**Figure 1:**
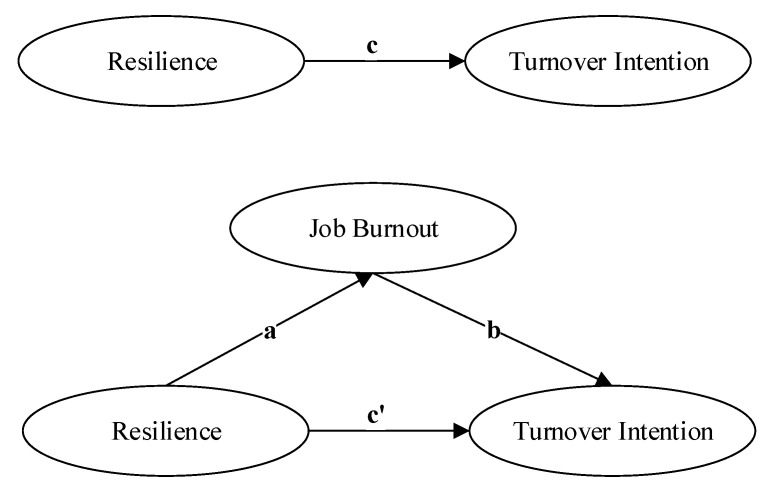
Research model.

The specific hypothetical model is shown in Figure 1 below.

## 2. Materials and Methods

### 2.1. Procedure and Participants

In order to determine the relationship between resilience, job burnout, and turnover intention, a cross-sectional survey was conducted among 500 high school teachers in Jiangsu Province, China, from November 2020 to January 2021. The main experimental procedure was to send the revised scales of resilience, job burnout, and turnover intention to teachers through the online teaching platform of the education department in order to measure their levels. At the same time, this method also ensured that all participants were high school teachers and had online teaching experience. A total of 2463 registered teachers (721 high school teachers) were participating in online teaching on this platform, so the sample was accepted when high school teachers accounted for more than 50% of the respondents [48]. At the same time, was considered reasonable that the results of this research sample would conform to the normal distribution. The reason for adopting an online survey was that it is difficult for researchers to collect data in person due to the epidemic situation. All questionnaires informed participants about the purpose of the questionnaire at the beginning. All participants were high school teachers who volunteered to participate in the survey. After completing the questionnaire, they received a thank-you gift of 5 yuan. A total of 492 (98.40%) questionnaires were collected. The preliminary analysis of the returned questionnaires excluded blank questions and significant pattern (i.e., 111111) questionnaires showing false participation [49]. Overall, 449 questionnaires (89.80%) were passed on for further data analysis. Among the 449 high school teachers in this study, 118 were male (26.28%) and 331 female (73.72%). The average age of participants was 36.70 years (SD = 2.31).

### 2.2. Ethical Consideration

This study was conducted following the Declaration of Helsinki (2002) and the Measures for Ethical Review of Biomedical Research Involving Humans of the Ministry of Health, China. The protocol was approved by the Ethics Committee of Nanjing Normal University.

### 2.3. Measures

#### 2.3.1. Resilience Scale

Resilience was assessed with the scale devised by Connor and Davidson (2003) after revision [50]. There were 25 items and 3 dimensions: confidence, optimism, and strength (see Table A1 in Appendix A). A five-point score was used, ranging from 1 to 5, where 1 meant very inconsistent and 5 meant very consistent. In this study, the Cronbach’s alpha coefficient of the scale was 0.96 and the three-dimensional coefficient was 0.66–0.93, which indicated good reliability and suitability for use with Chinese high school teachers.

#### 2.3.2. Job Burnout Scale

Job burnout was assessed using the scale devised by Maslach (1981) [51]. The original scale has 25 items, while the shorter scale after localization revision had 10 items and 3 dimensions: emotional exhaustion, depersonalization, and low personal accomplishment (see Table A2 of Appendix A). A five-point score was used, ranging from 1 to 5, where 1 meant very inconsistent and 5 meant very consistent. In this study, the Cronbach’s alpha coefficient of the scale was 0.91 and the three-dimensional coefficient was 0.71–0.87, which indicated good reliability and suitability for use with Chinese high school teachers.

#### 2.3.3. Turnover Intention Scale

Turnover intention was assessed using the scale devised by Price (1981) after revision [52]. There were four items (see Table A3 of Appendix A). A five-point score was used, ranging from 1 to 5, where 1 meant very inconsistent and 5 meant very consistent. In this study, the Cronbach’s alpha coefficient of the scale was 0.84, which indicated good reliability and suitability for use with Chinese high school teachers.

### 2.4. Data Processing

In order to determine whether the measurement had satisfactory psychological measurement attributes, SPSS 25.0 (IBM, New York, NY, USA) was used to analyze the data and test the common method deviation, and the Cronbach’s alpha coefficient was used to evaluate the reliability of the scale. Secondly, descriptive statistics were used to analyze the data distribution, so as to judge whether the sample distribution was suitable for the next analysis. Thirdly, the correlation among the three variables was analyzed to judge whether the model could be constructed. Finally, a structural equation model was constructed by Amos 24.0 (IBM, New York, NY, USA) to explore the relationship between resilience, job burnout, and turnover intention.

## 3. Results

### 3.1. Common Method Deviation Test

Several test scales were used in this study and all of them were conducted in a unified way. The content of the questionnaire, the characteristics of the participants, and the environment of the tests could have caused covariation between the efficacy standard and the prediction, which could have led to deviation of the research results [53]. In order to effectively judge the existence of common method deviation, a Harman single factor test was adopted in this study, and exploratory factor analysis was undertaken for all items. In this analysis, when the eigenvalue root was greater than 1, the variance explained by the first factor was 14.56% < 40%. Therefore, there was no serious common method deviation among the variables in this study.

### 3.2. Descriptive Statistic

In this study, resilience, job burnout, and turnover intention were analyzed utilizing means, standard deviations, maximum values, and minimum values, as shown in Table 1.

In terms of resilience, the average resilience was significantly higher than the median, indicating that the resilience level of high school teachers was at a high level. Confidence, optimism, and strength were higher than the median, which indicates that high school teachers have a strong level of resilience in all aspects and can cope well with difficulties.

In terms of turnover intention, the average turnover intention of teachers was very close to the median, which indicates that the turnover intention of high school teachers is not favorable. In China, due to the particularity of the college entrance examination, high school teachers face great pressure with regard to students’ scores and future studies.

Similar to the turnover intention, the average value of teachers’ job burnout was very close to the median value, which also indicated that the job burnout prospects of high school teachers are not favorable. As high school teachers are faced with heavy teaching tasks, it is difficult to adjust quickly even if they have strong psychological qualities.

### 3.3. Correlation Analysis

The Pearson correlation coefficient can effectively test the correlation between two variables [54]. In this study, the Pearson correlation coefficient was used to examine the relationships between resilience, job burnout and turnover intention and among all dimensions. The specific results are shown in Table 2.

As shown in Table 2, all dimensions showed a significant correlation. Among them, resilience significantly negatively correlated with turnover intention (*p* < 0.01) and job burnout (*p* < 0.01). Turnover intention significantly positively correlated with job burnout (*p* < 0.01). All three dimensions of resilience significantly predicted resilience positively. All three dimensions of job burnout also significantly predicted job burnout positively.

### 3.4. Regression Analysis

As there was a high correlation between resilience and various dimensions, it would have been easy to end up with multicollinearity problems if three variables were directly selected for multivariate stepwise regression analysis and the conditions for applying multivariate linear regression were not valid. Therefore, in order to eliminate this problem, this study adopted a simple linear regression analysis of pairwise variables, and the specific results were divided into the following three categories.

#### 3.4.1. Regression Analysis of Resilience and Turnover Intention

In this study, we set the independent variables as resilience with three dimensions and the dependent variable as turnover intention. Through regression analysis we obtained the results shown in Table 3.

From the results in Table 3, it can be seen that the regression model of resilience and turnover intention was F = 785.121 (*p* < 0.001) in the overall test. Resilience, with three dimensions, could effectively explain the variance of turnover intention as 33.5%, 31.1%, 32.1%, and 14.7%, respectively. After standardization, it was found that resilience could effectively predict turnover intention negatively.

#### 3.4.2. Regression Analysis of Resilience and Job Burnout

In this study, we set the independent variables as resilience with three dimensions and the dependent variables as three dimensions of job burnout. Through regression analysis we obtained the results shown in Table 4.

From the results in Table 4, it can be seen that the F values of resilience and job burnout in the regression model corresponded to *p* < 0.001, which indicated that they were at a significant level in all dimensions. At the same time, from the coefficients of the regression model, it was concluded that resilience could effectively predict job burnout negatively.

#### 3.4.3. Regression Analysis of Job Burnout and Turnover Intention

In this study, there was a significant positive correlation between job burnout and its various dimensions and turnover intention, and the correlation between the various dimensions was below 0.7. Therefore, the forced entry variable method was adopted and emotional exhaustion, depersonalization, and low personal accomplishment were put into the regression model one by one to investigate the importance levels of the variables. The analysis results are shown in Table 5.

From the results in Table 5, it can be seen that the three dimensions of job burnout and the F value of the regression model of turnover intention corresponded to *p* < 0.001, which indicated that all three variables had significant positive predictive effects on turnover intention. The multiple regression coefficient for the turnover intention was 0.581, and the multiple decision coefficient was 0.326, which together could effectively explain the 32.6% variance in turnover intention. Among these three dimensions, emotional exhaustion was the most predictive factor for turnover intention with an explanatory variance of 29.2%, followed by depersonalization with an explanatory variance of 1.9%, and the worst predictive factor was low personal accomplishment with 1.5%.

### 3.5. Mediating Effect Analysis

In this study, resilience and job burnout belonged to latent variables, and the scores of the various dimensions of job burnout simply ended up being meaningless and could not be measured directly. If explicit variables are used to analyze the mediating effect between variables, the proportion and composite reliability of the actual mediating effect will be underestimated. Therefore, in this study, the nonparametric percentile bootstrap method with deviation correction was adopted, and a structural equation model was established with latent variables to test the mediating effect. Furthermore, this study assumed that job burnout was an intermediary variable between resilience and turnover intention. In the regression analysis, it could be seen that resilience had a significant negative predictive effect on job burnout and turnover intention and that coefficients a and c were significant. Additionally, turnover intention could significantly predict turnover intention positively and coefficient b was significant. Once the first three conditions were met, the next step was to take the latent variable resilience as the independent variable, the latent variable job burnout as an intermediate variable, and the explicit variable turnover intention as the dependent variable, and then establish a structural equation model and verify whether a∗b = 0 through the nonparametric percentile bootstrap method with deviation correction.

**Figure 2:**
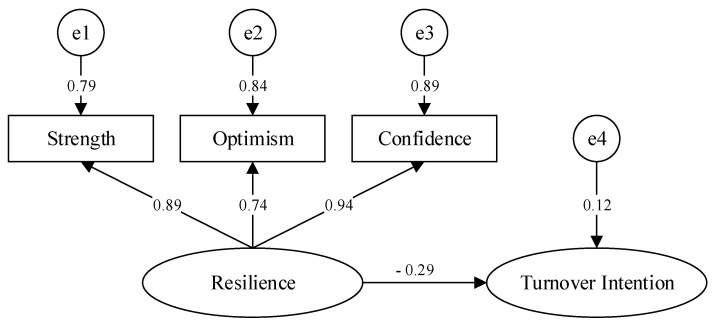
Direct effect model.

It can be seen from Table 6 that each index of the two models was greater than 0.90, which indicates that the two models had good fitness. From the RMSEA, the adaptation degree of the main effect model and the intermediary effect model also showed good fitness. The X^2^/df of the main effect model and the intermediate model also met the normal values. Therefore, according to the multivariate judgment criterion of the structural equation model, it can be concluded that the theoretical model of the main effect and the intermediary effect in this study had a good degree of adaptation and a good degree of conformity with the actual data of the samples. In addition, this study constructed a direct effect model of resilience and turnover intention, as shown in Figure 2.

At the same time, when constructing the mediation model, this study adopts variance maximum likelihood method and the deviation corrected percentile Bootstrap test for parameter estimation and mediation effect test of structural equation model. After repeated sampling 5000 times, 95% confidence interval is calculated. The specific results are shown in Table 7.

**Figure 3:**
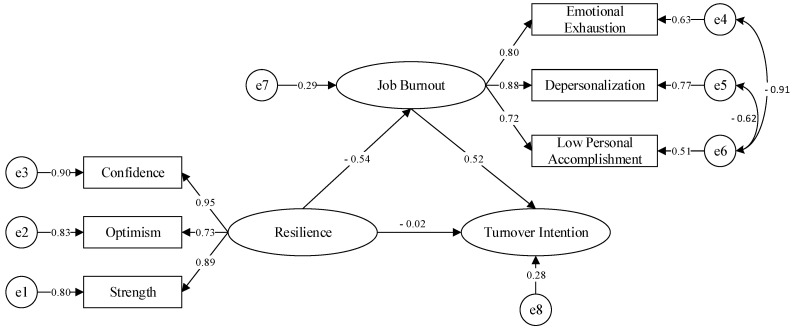
Mediating effect model.

It can be seen from Table 6 that resilience has a direct effect on turnover intention (λ = −0.294, *p* < 0.001), resilience has a direct effect on job burnout (λ = −0.539, *p* < 0.001), and resilience has an indirect effect on turnover intention through job burnout. After adding job burnout, the influence of resilience on turnover intention changed from significant to insignificant. The confidence interval of 95% tested by the nonparametric Bootstrap method with deviation correction contains 0, which indicates that job burnout plays a complete mediating role between resilience and turnover intention, and the mediating effect accounts for 95.5% of the total effect. Therefore, this study constructs the mediation model as shown in Figure 3 below.

## 4. Discussion

### 4.1. The Relationship between Resilience and Turnover Intention

Resilience and its dimensions of confidence, strength, and optimism had a significant negative correlation with turnover intention, and resilience could significantly predict turnover intention negatively. That is to say that the higher the resilience levels of high school teachers, the weaker their turnover intention is, and that resilience level affects turnover intention.

According to the turnover model of Price-Mueller [55], personal emotion, job satisfaction, job stress, and other factors have an impact on turnover intention. Teachers with good resilience have strong adaptability and a high level of response. They have more positive emotions and less negative emotions in their work, and can actively cope with work pressure and enhance their professional self-confidence [56]. With the improvement of teachers’ working ability, it is easier to get better development opportunities in schools, and school leaders are entrusted with more and more important tasks [57]. Teachers’ needs are consistently met, their job satisfaction is higher, and they show a greater sense of value and belonging in their work at school, which can help teachers seek long-term stable development in school, and thus result in reduced turnover intention [58]. However, high school teachers with poor resilience have problems such as a weak ability to adapt to the environment, passive avoidance of challenges, passive coping with tasks, lack of job competence, and difficulty in building self-confidence. As a result, their interpersonal relationships with colleagues easily deteriorate, and they are more likely to have negative emotions such as loneliness, hostility, and feelings of meaninglessness, and worthlessness. They are confused and question about how long their teaching career can last, and thus they are more likely to have increased turnover intention.

### 4.2. The Relationship between Resilience and Job Burnout

Resilience and its dimensions had a significant negative correlation with job burnout dimensions, and resilience could significantly predict job burnout negatively. That is to say that the lower the resilience level of high school teachers, the more serious the job burnout is, and that resilience can affect job burnout.

A new model for monitoring resilience holds that everyone has the nature to keep a biological, psychological, and spiritual balance. When this balance is broken by external pressure, people will mobilize various protective factors to restore the balance [59]. As an important psychological and social resource, resilience includes internal positive psychological resources and external social support resources that can help individuals successfully cope with difficulties, adapt to pressures, and develop [60]. Teachers with high resilience can adjust themselves and overcome difficulties when facing the problems brought by the epidemic situation and the pressure of job performance. They have optimistic, confident, positive, and healthy emotions, which can help them stay away from the emotional distress of job burnout to a certain extent. In addition, the school is a collective that pays great attention to interpersonal relationships [61,62]. The completion of many teaching tasks requires not only the coordination of teaching methods among high school teachers but also communication with students and parents. In the process of actively overcoming difficulties and completing tasks, teachers with good resilience maintain good interpersonal relationships with colleagues, students, and parents. Teachers’ interpersonal relationships are often better while their sense of work efficiency and competence is greatly improved, which can alleviate the interpersonal troubles caused by the disintegration of personality in burnout and the problem of low self-worth caused by the reduction of the sense of accomplishment [23].

### 4.3. The Relationship between Job Burnout and Turnover Intention

All dimensions of job burnout were positively correlated with turnover intention, and all dimensions of job burnout could positively predict turnover intention. That is to say that the more serious job burnout is, the more serious turnover intention is, and that job burnout affects turnover intention.

From the regression analysis, it can be seen that emotional exhaustion was the strongest predictor of turnover intention and low personal accomplishment was the weakest among the three dimensions of job burnout. After low personal accomplishment was included in the regression equation, the standard regression coefficient was significant, which can explain to some extent why high school teachers’ overall job burnout and turnover intention are serious.

However, scholars are in disagreement about the relationship between job burnout and turnover intention [63]. Yang and Zhang (2015) [64] found that the relationship between emotional exhaustion, depersonalization, and turnover intention was significant, while the relationship between low personal accomplishment and turnover intention was not significant, which was different from the conclusion of this study. The reasons for this difference may be related to the differences in sample size and research objects.

### 4.4. Mediating Effect

Job burnout played a complete mediating role between resilience and turnover intention, and the mediating effect accounted for 95.5% of the total effect; that is, resilience had a full effect on turnover intention through job burnout, while resilience had almost no direct effect on turnover intention (1.9%), which had to be instead transmitted through job burnout, this being the only path between resilience and turnover intention. Tziner (2015) [65] found that, beyond the assumed direct relationships, burnout partially mediated between work stress and work satisfaction, and work satisfaction partially mediated the relationship between burnout and turnover intentions of hospital physicians.

Resilience has a positive role in alleviating job burnout, but the outbreak of the epidemic has brought unprecedented changes to teachers’ work. Burnout now not only has a deep impact on teachers’ physical and mental health, job performance, job satisfaction, and turnover intention, but also has an increasingly wider scope [51]. Job burnout is becoming more and more important, and it has a greater impact on turnover intention, which needs to be paid great attention.

Complete mediation is established in theory, but it is extremely rare in reality. The test of mediation effects in this study was carried out under the condition of artificially excluding the influence of other variables, only verifying the influences of resilience and job burnout on turnover intention, without considering the possibility of other mediation variables at all. The reality is complex, and the factors are various. Other variables that have mediating effects between resilience and turnover intention may exist in reality.

## 5. Conclusions

After investigating and analyzing the relationships between the resilience, job burnout, and turnover intention of 449 Chinese high school teachers, this study drew the following two main conclusions.

On the one hand, despite the inconvenience and challenges caused by the outbreak of the epidemic, high school teachers’ resilience levels remain high compared with the strict problems of job burnout and turnover intention. At the same time, resilience had a significant negative predictive effect on job burnout and turnover intention, which does not mean that we can ignore its existence. On the contrary, teachers should undergo mental health education and training to deal with all kinds of emergencies before entering the job, which can help teachers cope with emergencies encountered in both teaching and non-teaching situations. Furthermore, due to having previous experience of various adversities, teachers would show better psychological adaptation and effective adjustment measures when facing such events again.

On the other hand, job burnout had a significant positive predictive effect on turnover intention and played a completely mediating role between resilience and turnover intention. The most important implication of this result is that if teachers are not satisfied or are treated unfairly in their work, they are likely to experience resistance toward their work. In addition, due to working from home for a long time, teachers cannot seek help and relieve psychological problems in time, so it is easy to experience job burnout. At this time, if school leaders and parents add an extra workload outside the scope of the job, this can easily lead to the problem of teachers leaving their posts, and these phenomena lead onto each other. Therefore, this result also indicates that future research should pay more attention to the causes of teachers’ job burnout and turnover intentions. In addition, when teachers are faced with such psychological problems, job burnout and turnover intention are the key points that high schools should pay attention to during the epidemic, as they have a great impact on the effective development of high school teaching.

## 6. Suggestions

Briefly, the conclusion of this study provides important inspiration for the educational management of schools and the professional development of high school teachers. Therefore, this study puts forward the following suggestions for consideration in practice.

On the one hand, the findings of this study can provide the theoretical basis for school leaders to reduce high school teachers’ turnover intentions. School leaders can alleviate teachers’ job burnout by training and improving teachers’ resilience, which can achieve the goal of reducing teachers’ turnover intentions. People with high levels of resilience can not only recover quickly after encountering adversity but also become strong in the process, which indicates that teachers should have better resilience. When teachers have good resilience, they can further guide and cultivate students’ resilience and help them obtain a better education. In the epidemic situation in particular, teachers can strengthen their levels of resilience through self-learning and further improve the levels of students’ resilience through online resilience training courses.

On the other hand, this study confirmed that job burnout plays an intermediary role between high school teachers’ resilience and turnover intentions, which indicates that relieving job burnout can reduce teachers’ turnover intentions. Specifically, school leaders can take preventive measures to intervene in teachers’ job burnout from four perspectives: society, school, family, and individual. First of all, society should form reasonable expectations for teachers and should not completely attribute social and family responsibilities to schools and teachers. Secondly, schools need to create a high-quality working environment and a good interpersonal atmosphere, such as by providing teachers with office equipment with excellent performance, appropriately reducing teachers’ workload, and avoiding arranging administrative work unrelated to education. Furthermore, teachers’ families need to give teachers more understanding and care, especially when they are working at home during the epidemic, and teachers’ psychological burdens and anxiety can be reduced through family entertainment activities. Finally, teachers need to have time to study and enhance their professional qualities. Job burnout is often a kind of “ability panic”. Teachers need to continue to study in order to adapt to the pressure of the social environment.

## 7. Limitations

Firstly, although the sample size in this study was representative and theoretically explanatory, the sample was only from one province in China. The sample representation thus needs to be strengthened due to the lack of samples from other provinces. Therefore, more participants from different regions are needed to generalize the research results across a wider range.

Secondly, the scales used in the study were only intended for the general population. Although this study revised the scales, judging whether they can be applied well to teachers as a group or even specifically to high school teachers requires further verification by considering the specificity of teachers’ work.

Thirdly, the online teaching platform of the education department was used to distribute the questionnaire. Although this approach was able to obtain strong support and monitoring at the government level and made it possible to put forward specific requirements for participants, quality control still needs to be strengthened because the questionnaire was not efficient enough.

## Figures and Tables

**Table 1:** Descriptive statistics.

Variable	Min	Max	Average	SD
Resilience	25	125	88.00	13.98
Confidence	8	40	29.65	4.81
Optimism	4	20	13.81	2.42
Strength	13	65	44.54	7.70
Turnover intention	4	20	10.14	3.24
Job burnout	10	50	26.89	6.89
Emotional exhaustion	5	25	13.54	3.96
Depersonalization	3	15	8.06	1.63
Low personal accomplishment	2	10	6.01	1.21

**Table 2:** Descriptive statistic.

Variables	1	2	3	4	5	6	7	8	9
1. Resiliense	1								
2. Confidence	0.938 **	1							
3. Optimism	0.780 **	0.701 **	1						
4. Strength	0.966 **	0.846 **	0.755 **	1					
5. Turnover intention	−0.283 **	−0.289 **	−0.216 **	−0.261 **	1				
6. Job burnout	−0.473 **	−0.461 **	−0.379 **	−0.443 **	0.485 **	1			
7. Emotional exhaustion	−0.425 **	−0.391 **	−0.355 **	−0.408 **	0.438 **	0.935 **	1		
8. Depersonalization	−0.450 **	−0.470 **	−0.330 **	−0.412 **	0.480 **	0.870 **	0.691 **	1	
9. Low personal accomplishment	−0.354 **	−0.360 **	−0.290 **	−0.322 **	0.333 **	0.774 **	0.584 **	0.632 **	1

Note: **, *p* < 0.05.

**Table 3:** Descriptive statistics.

Independent Variable	Dependent Variable	R	R^2^	F	B	β
Resilience	Turnover Intention	0.562	0.335	785.121 ***	−9.871	−0.562
Confidence		0.311	0.092	153.257 ***	−0.223	−0.311
Optimism		0.321	0.899	164.265 ***	−0.161	−0.321
Strength		0.147	0.026	38.921 ***	−0.271	−0.147

Note: ***, *p* < 0.001.

**Table 4:** Descriptive statistics.

Independent Variable	Dependent Variable	R	R^2^	F	B	β
Resilience	Emotional exhaustion	0.322	0.141	248.361 ***	−3.121	−0.322
Confidence		0.381	0.103	256.312 ***	−0.377	−0.381
Optimism		0.352	0.142	245.981 ***	−0.268	−0.352
Strength		0.241	0.147	91.925 ***	−0.577	−0.241
Resilience	Depersonalization	0.396	0.173	331.021 ***	−3.041	−0.396
Confidence		0.448	0.212	409.658 ***	−0.413	−0.448
Optimism		0.373	0.129	248.742 ***	−0.277	−0.373
Strength		0.405	0.161	299.188 ***	−0.234	−0.405
Resilience	Low personal accomplishment	0.476	0.253	498.899 ***	−4.451	−0.476
Confidence		0.498	0.264	543.234 ***	−0.502	−0.498
Optimism		0.477	0.203	454.872 ***	−0.333	−0.477
Strength		0.402	0.144	274.787 ***	−0.031	−0.402

Note: ***, *p* = 0.000.

**Table 5:** Descriptive statistics.

Independent Variable	Dependent Variable	R	R^2^	ΔR^2^	F	ΔF	B	β
Emotional exhaustion	Turnover intention	0.501	0.292	0.292	159.085 ***	698.213 ***	0.389	0.434
Depersonalization		0.573	0.311	0.019	95.765 ***	35.326 ***	0.167	0.172
Low personal accomplishment		0.581	0.326	0.015	69.331 ***	20.405 ***	0.106	0.113

Note: ***, *p* < 0.001.

**Table 6:** Descriptive statistics.

Model	X^2^/df	RMSEA	GFI	AGFI	IFI	CFI
Main effect	1.131	0.001	1.000	0.998	0.999	0.999
Mediating effect	2.423	0.073	0.982	0.973	0.987	0.976

**Table 7:** Fitting index of the regression model.

a	b	c	c’	ab/c	95% Confidence Interval	
					LLCI	ULCI
−0.539 ***	0.521 ***	−0.294 ***	−0.019	0.955	−0.084	0.063

Note: ***, *p* < 0.001.

## Data Availability

The data that support the findings of this study are available from the corresponding author. Restrictions apply to the availability of these data, which were used under license for this study. Data are available from the authors with the permission of Nanjing Normal University.

## Appendix A

**Table A1:** Resilience scale.

No.	Items
**1**	I can adapt to changes.
**2**	I am in a close and safe relationship.
**3**	I am proud of my achievements.
**4**	I work hard to achieve my goal.
**5**	I feel in control of my life.
**……**	
**21**	I won’t be discouraged by failure.
**22**	I often recover quickly after hardship or illness.
**23**	I know where to ask for help.
**24**	I can concentrate and think clearly under pressure.
**25**	I like to take the lead in solving problems.

**Table A2:** Job burnout scale.

No.	Items
**1**	I am always listless when I wake up every day and listless when facing work.
**2**	I feel very tired after a day’s work.
**3**	My work often bores me.
**4**	Work often requires me to consume too many emotional resources.
**5**	I am confused about my present career prospects.
**6**	I lost my enthusiasm for my current job.
**7**	I don’t think the present job is of any value.
**8**	I feel that I am not qualified for my present job.
**9**	Leaders often put me in charge of some unimportant things.
**10**	I often have no confidence in the tasks assigned to me by my leaders.

**Table A3:** Turnover intention scale.

No.	Items
**1**	I don’t want to leave my present unit.
**2**	I plan to make long-term career development in this unit.
**3**	I often get bored in my current job and want to change to a new unit.
**4**	I will probably leave my present unit in the next six months.
